# On the Nature of the Green Alvine Evacuations of Children

**Published:** 1846-01-01

**Authors:** 


*On the nature of the Green Alvine evacuations of Children.*—The green evacuations so common in the diseases of children, are usually supposed to denote the presence of bile in combination with an acid. Dr. Golding Bird, of London, however, has subjected a specimen to careful chemical analysis, and found only traces of bile present. He regards the green color of the stools which the English writers compare to "chopped spinage," as "depending on the presence of modified blood, rather than on an excess of bile." He says, "believing that the green stools alluded to are but a form of melœna, I have often closely questioned the nurses of children voiding them, regarding the appearance of the development of the green color, and have almost constantly been told that streaks, and even clots of blood had been observed. I regard, then, the presence of green stools as indicative, not of a copious secretion of bile, but of a congested state of the portal system, in which blood is exuded very slowly, and in small quantities, so as to allow of the color being affected by the gases and secretions present in the intestines; a state of things capable of readily ending in melœna, in which the effusion of blood is so copious and sudden, as not to give time for the occurrence of the changes alluded to."—*Bulletin of Med. Science.*

